# Creating and parameterizing patient-specific deep brain stimulation pathway-activation models using the hyperdirect pathway as an example

**DOI:** 10.1371/journal.pone.0176132

**Published:** 2017-04-25

**Authors:** Kabilar Gunalan, Ashutosh Chaturvedi, Bryan Howell, Yuval Duchin, Scott F. Lempka, Remi Patriat, Guillermo Sapiro, Noam Harel, Cameron C. McIntyre

**Affiliations:** 1 Department of Biomedical Engineering, Case Western Reserve University, Cleveland, Ohio, United States of America; 2 Department of Radiology, University of Minnesota, Minneapolis, Minnesota, United States of America; 3 Center for Neurological Restoration, Cleveland Clinic, Cleveland, Ohio, United States of America; 4 Research Service, Louis Stokes Cleveland Veterans Affairs Medical Center, Cleveland, Ohio, United States of America; 5 Department of Electrical and Computer Engineering, Duke University, Durham, North Carolina, United States of America; 6 Department of Biomedical Engineering, Duke University, Durham, North Carolina, United States of America; 7 Department of Computer Science, Duke University, Durham, North Carolina, United States of America; Oslo Universitetssykehus, NORWAY

## Abstract

**Background:**

Deep brain stimulation (DBS) is an established clinical therapy and computational models have played an important role in advancing the technology. Patient-specific DBS models are now common tools in both academic and industrial research, as well as clinical software systems. However, the exact methodology for creating patient-specific DBS models can vary substantially and important technical details are often missing from published reports.

**Objective:**

Provide a detailed description of the assembly workflow and parameterization of a patient-specific DBS pathway-activation model (PAM) and predict the response of the hyperdirect pathway to clinical stimulation.

**Methods:**

Integration of multiple software tools (e.g. COMSOL, MATLAB, FSL, NEURON, Python) enables the creation and visualization of a DBS PAM. An example DBS PAM was developed using 7T magnetic resonance imaging data from a single unilaterally implanted patient with Parkinson’s disease (PD). This detailed description implements our best computational practices and most elaborate parameterization steps, as defined from over a decade of technical evolution.

**Results:**

Pathway recruitment curves and strength-duration relationships highlight the non-linear response of axons to changes in the DBS parameter settings.

**Conclusion:**

Parameterization of patient-specific DBS models can be highly detailed and constrained, thereby providing confidence in the simulation predictions, but at the expense of time demanding technical implementation steps. DBS PAMs represent new tools for investigating possible correlations between brain pathway activation patterns and clinical symptom modulation.

## 1. Introduction

Deep brain stimulation (DBS) is an established therapy for the treatment of movement disorders (e.g. essential tremor, Parkinson’s disease (PD), and dystonia) and shows promise for the treatment of epilepsy and neuropsychiatric diseases (e.g. obsessive compulsive disorder, Tourette syndrome, and depression) [1]. Despite the growing clinical use of DBS, there is a paucity of knowledge on the neural response to the applied voltage distribution, and correlations linking the modulation of different brain pathways with clinical outcomes are lacking. Pathway-activation models (PAMs) are new scientific tools designed to help to address those knowledge gaps.

The motivation for creating PAMs comes from the clinical observation that accurate placement of the electrode within the target is a major determinant of therapeutic outcomes in DBS interventions [2–4]. However, a clear scientific definition of the “target” for each DBS therapy has been somewhat elusive. Experimental and theoretical data suggest that axons are the most excitable neural elements to extracellular electrical stimulation [5,6], and a primary effect of DBS is the generation of action potentials in axons [7,8]. Thus, irrespective of the neurological disorder under consideration, a growing consensus suggests that the target of the stimulation is likely to be axonal in nature [4,9]. However, the specific axonal pathways that are the explicit therapeutic targets for DBS are still under debate.

Given that a basic purpose of diffusion-weighted imaging (DWI) is to characterize axonal pathways in the brain, a burgeoning field of DBS research is now using DWI-based tractography to better understand the activated pathways [10,11]. Numerous clinical studies have recently conducted tractography from voxels near DBS electrode contacts to identify potential axonal pathways that may be stimulated in disorders such as PD [12], essential tremor [13], depression [14], and epilepsy [15]. However, studies of this type commonly ignore the underlying biophysics of electrical stimulation when attempting to identify activated pathways. PAMs represent a methodology to explicitly calculate the axonal response to DBS, as well as its dependence on a number of factors that include: 1) the electrode configuration, 2) the shape, duration, and frequency of the applied stimuli, 3) the electrical conduction properties of the brain tissue medium, 4) the geometry and trajectory of the axons, and 5) the membrane biophysics of the axons.

We propose that accurate assessment of axonal activation requires modeling the direct application of the DBS voltage distribution on anatomically and biophysically accurate models of axons. Chaturvedi et al. [16] and Lujan et al. [17,18] demonstrated our first attempts at creating the conceptual basis of PAMs. These studies used medical images to locate the DBS electrode and model the voltage distribution generated in the patient’s head. Then tractography was used to define the location and trajectory of axonal pathways surrounding the electrode. Finally, the DBS voltage distribution was used to stimulate cable models of individual axons. However, these first generation PAMs had very difficult software integration hurdles that exceeded what would be realistic for use in larger scale clinical analyses, as well as technical limitations in the volume conductor electric field models. Therefore, we worked to develop an improved workflow for constructing PAMs, and implemented numerous model parameterization steps that improve the detail and accuracy of the simulations. This manuscript describes how each step of the workflow comes together to create a PAM.

We present an example patient-specific PAM of unilateral subthalamic DBS that characterizes stimulation of two corticofugal pathways: 1) internal capsule fibers of passage, and 2) the hyperdirect pathway. Layer V pyramidal neurons send projections via the internal capsule to the brainstem and spinal cord. Of these projections, 5–10% give off a collateral to the subthalamic nucleus (STN) and are collectively known as the hyperdirect pathway [19–21]. Electrical [22,23] and optogenetic [9,24] stimulation of the hyperdirect pathway has been directly linked to therapeutic benefit in rodent models of PD. In addition, human experiments have supported the hypothesis that DBS of the hyperdirect pathway is related to symptom relief [25,26]. In contrast, direct activation of internal capsule fibers of passage is known to generate muscle contraction side effects [27]. Therefore, we use our PAM example to demonstrate the different DBS recruitment characteristics of these two clinically relevant pathways.

## 2. Materials and methods

### 2.1. Ethics statement

Collection of all patient data for this study was approved by the University of Minnesota Institutional Review Board (IRB). The patient provided informed written consent prior to participating in the research and this consent procedure was approved by the IRB.

### 2.2. Patient data

The imaging data was acquired from a 67-year old right-handed male diagnosed with PD for ~11 years. A Medtronic 3389 DBS lead was implanted in the left STN and connected to an Activa SC implantable pulse generator (IPG) (Medtronic, Minneapolis, MN). Using standard clinical programming procedures [28], the following therapeutic stimulation parameters were selected: monopolar configuration with contact 2 as the cathode and the IPG case as the anode, pulse amplitude of 1.7 V, pulse width of 60 μs, and pulse frequency of 130 Hz. His OFF medication, OFF stimulation motor subscore of the Unified Parkinson’s Disease Rating Scale was 31, and the ON medication, ON stimulation score was 14. The impedance measured by the IPG at contact 2 was 1450 Ω, which is the dynamic load of the circuit as defined at 70 μs into the stimulus pulse (Section E in S1 Text).

### 2.3. Workflow overview

**Fig 1:**
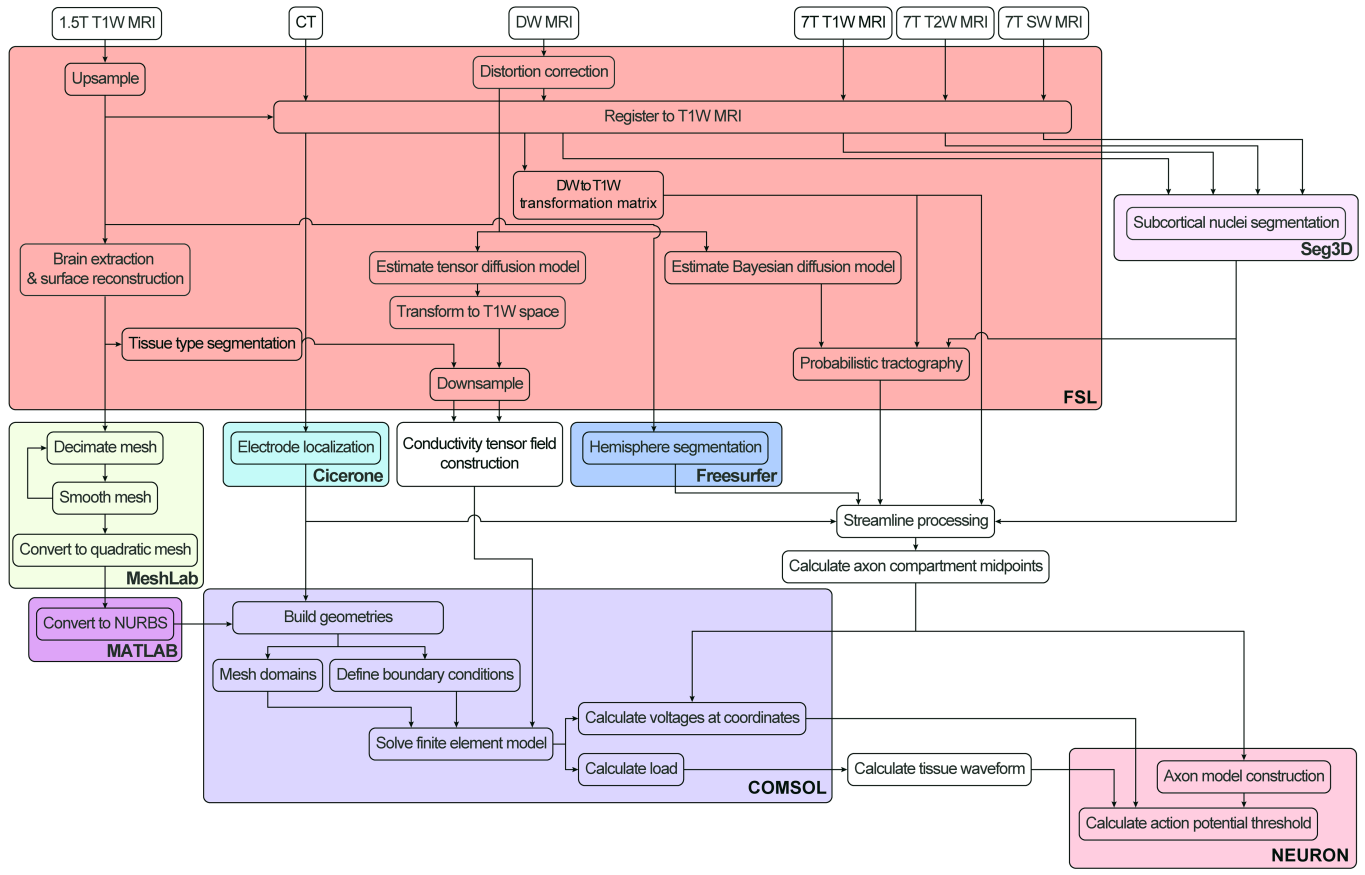
Scientific workflow for development of pathway-activation models. Color shading corresponds to the software program used for each step. Patient images are processed and tractography is performed in FSL (red). The finite element model is constructed and solved in COMSOL (purple). The axon model is constructed and the threshold stimulus amplitude for action potential generation is solved for in NEURON (pink). We automated many of the steps using custom MATLAB, Python, NEURON, and Bash scripts.

The general workflow required to create a PAM is outlined in Fig 1 and detailed in the following sections. First, we acquired, pre-processed, and co-registered the patient’s imaging data (Section 2.4 and Sections B and C in S1 Text). Second, we calculated the voltage distribution generated by the DBS electrode (Section 2.5). Third, we constructed multi-compartment cable axon models whose trajectories were based on tractography reconstructions of axonal pathways of interest near the DBS electrode (Section 2.6). Fourth, we used the DBS voltage distribution to stimulate the model axons and quantified their response (Section 2.7).

### 2.4. Image acquisition

The patient underwent pre-operative scanning on a 7T magnetic resonance imaging (MRI) system (Magnex Scientific, UK) at the Center for Magnetic Resonance Research (CMRR) at the University of Minnesota, using T1-weighted (T1W), T2-weighted (T2W), susceptibility-weighted (SW), and diffusion-weighted (DW) imaging (S1 Table and Section B in S1 Text). We also obtained a pre-operative T1W image on a 1.5T Siemens Magnetom Espree. A post-operative CT image was acquired on a Siemens Biograph64 Sensation approximately 1 month after the DBS surgery.

### 2.5. DBS voltage distribution

**Fig 2:**
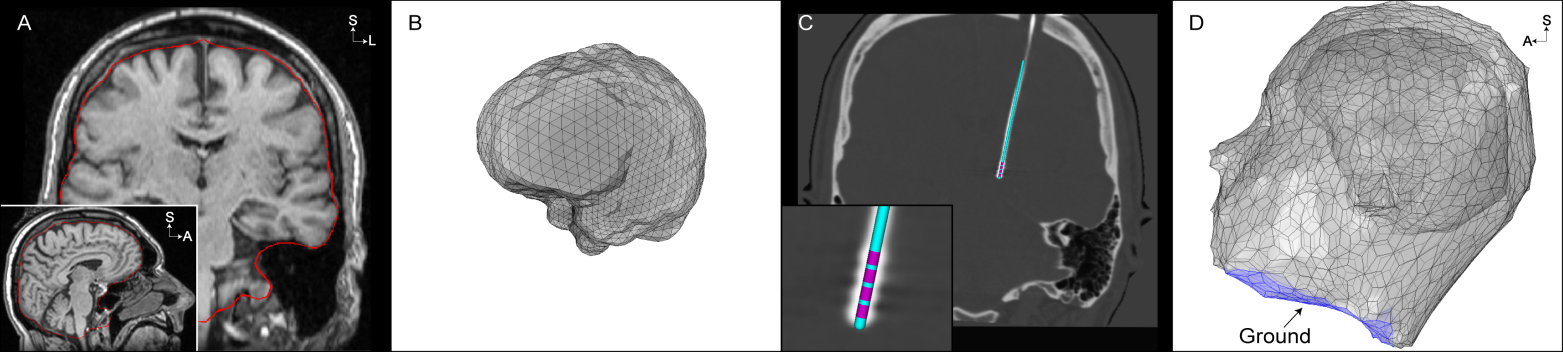
Finite element model boundaries. (A) The non-skull stripped 1.5T T1-weighted (T1W) image is used to extract the inner skull surface (red). (B) Inner skull surface mesh from (A) prior to any processing. (C) An oblique coronal view of the post-operative CT image, co-registered to the pre-operative T1W image, that is used to localize the four collinear electrode contacts. The inset shows the artifact of the 4 electrode contacts and a 3-dimensional rendering of the model Medtronic 3389 DBS electrode fit to the electrode artifact. (D) Domains of the finite element model, including the electrode, brain, and head. The neck region of the head surface mesh is set to 0 V under the monopolar configuration (blue).

**Fig 3:**
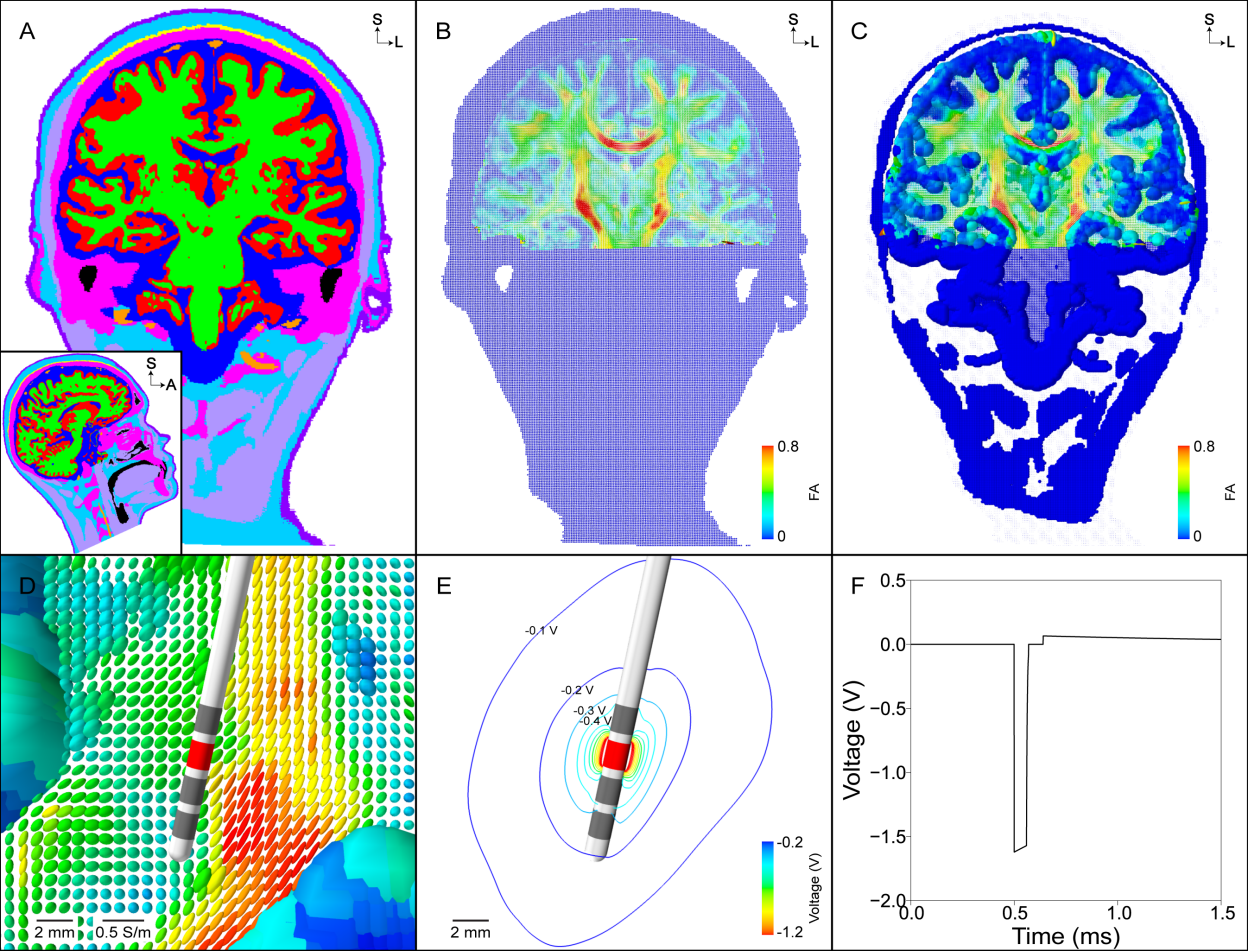
Finite element model and DBS voltage distribution. (A) Segmentation of the head into different tissue types (grey matter–red, white matter–green, cerebrospinal fluid–dark blue, muscle–light purple, tendon–yellow, bone–pink, fat–light blue, skin–dark purple, intervertebral disks–not visible, blood–orange, air–black). (B) Conductivity tensors within the head normalized by their volume. Anisotropic conductivity tensors are constructed within the brain using the eigenvectors of the diffusion tensors and a scalar mapping of the diffusion eigenvalues. Each tensor is colored according to its fractional anisotropy. (C) Same tensors from (B) but scaled so that the relative differences in conductivities can be visualized. (D) Zoomed view of tensors from (C) near the DBS electrode. (E) Isolines of the voltage distribution generated by a -1.7 V stimulus at contact 2. (F) The stimulus waveform at the electrode-tissue interface generated by the implantable pulse generator.

The voltage distribution generated by the DBS electrode varies both spatially and temporally in the tissue medium (Fig 2 and Fig 3). The conductance and permittivity of the tissue medium and electrode-tissue interface (ETI) affect the voltage distribution generated within the head. Temporally, the stimulus waveform generated by the IPG consists of a cathodic phase, interphase interval, passive recovery phase, and interpulse interval (Fig 3F). For a given set of stimulation parameters, we used a four-step approach to approximate the voltage distribution generated by the DBS electrode as a function of space and time (Eq 1) [29–32]:

$$\Phi (x,y,z,t)=\Phi (x,y,z,t=0)*A*V_{tissue}(t)$$ (1)

First, we calculated the static solution of the voltage distribution in the tissue medium, Φ(x,y,z,t = 0) (Fig 3E and Section 2.5.1). The voltage on the electrode surface was set to -1 V with respect to ground, which was defined at the base of the neck and set to 0 V (Fig 2D) [33]. Second, because the differential equation solved is linear, we scaled the voltage distribution by the stimulus amplitude, A, under investigation. Third, to account for the filtering effects of the IPG circuitry, lead wires, and the ETI on the DBS waveform “seen” by the tissue, we calculated the tissue voltage over time, V_tissue_(t), with an equivalent electrical circuit of the implanted DBS system (Fig 3F and S1 Fig and Section 2.5.2). Finally, the extracellular voltage distribution is scaled by the tissue waveform at each time step, Φ(x,y,z,t). This process is described in further detail in the following sections.

#### 2.5.1. Spatial characteristics

We calculated the voltage distribution generated in the tissue medium, Φ(x,y,z,t = 0), for monopolar cathodic stimulation delivered through contact 2. Laplace’s equation was solved using an electrostatic finite element model (FEM) in COMSOL. We constructed the FEM using the following five steps. First, we constructed volumes representing a Medtronic 3389 DBS electrode, an encapsulation layer surrounding the electrode, and domains of the brain and head. Each electrode contact was modeled as a cylindrical surface, with 1.5 mm length and 0.5 mm spacing between contacts. The length of the entire electrode shaft was 60 mm but did not pass outside the brain domain. We modeled the encapsulation layer with a radius of 0.5 mm along the entire length of the electrode shaft. Surface meshes representing the inner skull and outer head surfaces were constructed (Fig 2A and 2B and Section F in S1 Text) and imported into COMSOL to define volumes of the brain and head (Fig 2D).

Second, we defined a conductivity tensor field within the head (Section E in S1 Text). The tensor field outside of the brain was isotropic, and was anisotropic within the brain. Within the brain, we defined symmetric conductivity tensors using a load preservation approach that was based off of the patient-specific diffusion tensor data [31]. We defined the isotropic conductivity of the encapsulation layer so that the model impedance matched the clinically-measured impedance (S2 Fig). To do so, we varied the encapsulation layer conductivity between 0.05–0.2 S/m [34,35], and then calculated the model impedance by replicating the impedance measurements of the Medtronic programming device.

Third, we defined Dirichlet boundary conditions of -1 V at contact 2 and 0 V at the neck region of the head surface (Fig 2D). The inactive contacts were modeled using boundary conditions, and the electrode shaft (except for the contacts) and head surface (except for the neck region) were modeled as perfect insulators (Section G in S1 Text). Fourth, we generated a multi-resolution, tetrahedral volume mesh between the outer boundary of the DBS electrode and the inner boundary of the outer head (Section G in S1 Text). Fifth, we solved the model to calculate the voltage distribution, Φ(x,y,z,t = 0) (Fig 3E).

#### 2.5.2. Temporal characteristics

We calculated the temporal modulation of the voltage distribution using an equivalent electrical circuit model for voltage-regulated, monopolar stimulation (S1 Fig). The equivalent electrical circuit model included representations of the blocking capacitors (10 μF), extension wire and lead wire resistances (55 Ω), ETI with a double-layer capacitance and Faradaic resistance in parallel, and tissue resistance. The distributed values of the double-layer capacitance and Faradaic resistance of the ETI were 30 μF/cm^2^ and 150 Ωcm^2^, respectively, which equated to lumped values of 1.8 μF and 2.5 kΩ [36]. We ignored the tissue capacitance because the double-layer capacitance is approximately two orders of magnitude larger than the tissue capacitance [29,31]. The access resistance of our electrostatic FEM (i.e. tissue resistance) with contact 2 set as the working electrode was 1373 Ω (S2 Fig and Section E in S1 Text). A ‘parasitic’ capacitance (3 nF) and ‘parasitic’ resistance (20 kΩ) were included in parallel with the load of the DBS system so the voltage waveform generated across the tissue resistance had decay characteristics during the interphase interval that matched the measured waveform from the output of a Medtronic IPG (data not shown).

The voltage waveform generated across the tissue resistance (i.e. tissue waveform) was calculated for an applied rectangular pulse train (Fig 3F). For each pulse, the applied rectangular waveform consisted of a cathodic phase, interphase interval, passive recovery phase, and interpulse interval. The tissue waveform, V_tissue_(t), was calculated by applying Kirchhoff’s current law to the equivalent circuit model and using forward Euler numerical time integration to solve the ordinary differential equations. Finally, we scaled the extracellular voltage distribution, Φ(x,y,z,t = 0), by the tissue waveform, V_tissue_(t), at each time step to calculate the temporal aspects of the voltage distribution, Φ(x,y,z,t) (S1 Video and S2 Video).

### 2.6. Axon model

**Fig 4:**
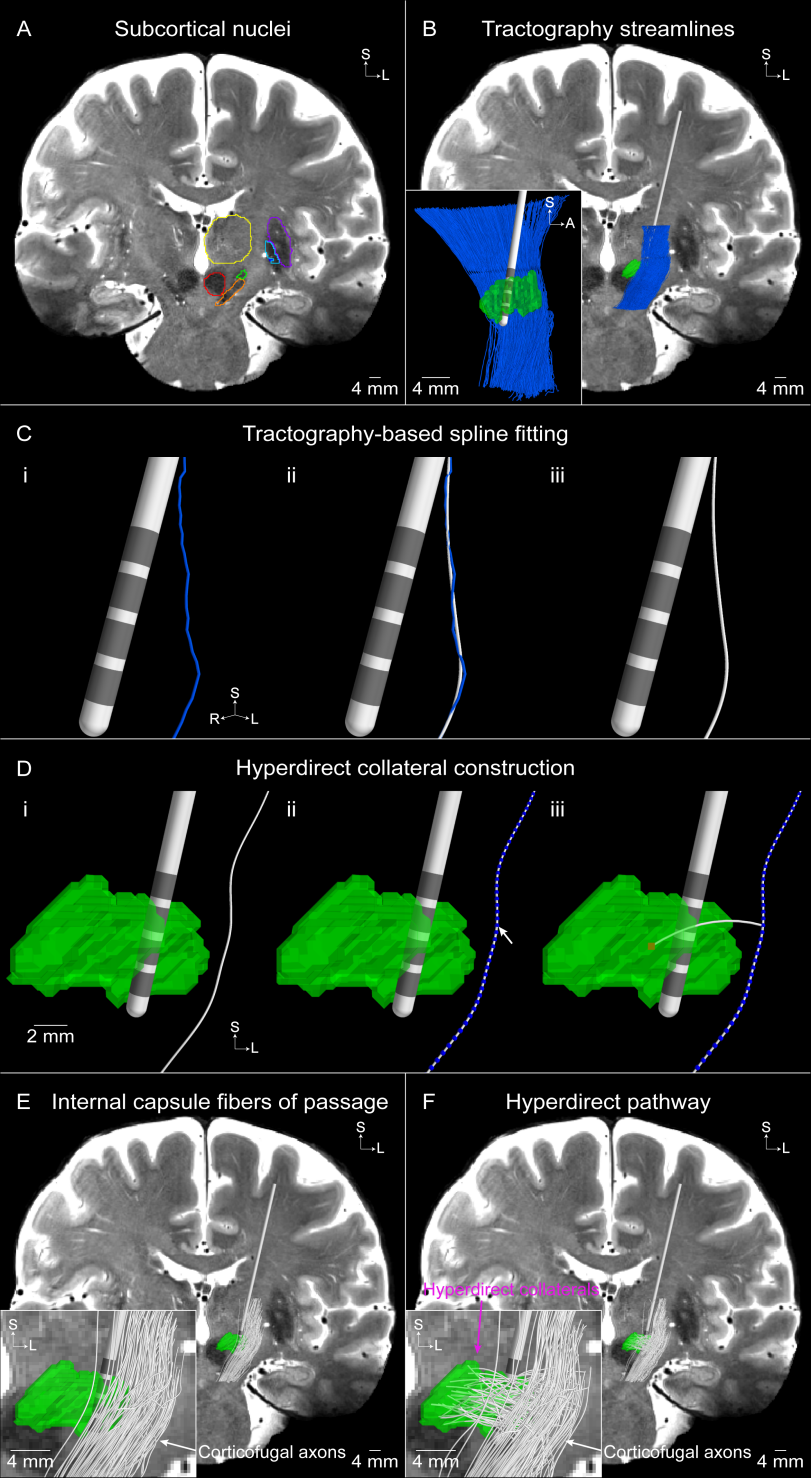
Tractography-based axon model of the hyperdirect pathway and internal capsule fibers of passage. (A) Subcortical nuclei outlined on the T2-weighted coronal image (subthalamic nucleus [STN]–green, substantia nigra–orange, red nucleus–red, thalamus–yellow, putamen–purple, globus pallidus externus–light blue, globus pallidus internus–dark blue). (B) Tractography-generated corticofugal streamlines. Inset is a sagittal view of the resulting streamlines. (C) A smoothing spline (white) is fit to an example tractography-generated streamline (blue). (D) The hyperdirect pathway axon is comprised of a collateral that branches off of a (i) corticofugal axon at a (ii) node of Ranvier (blue spheres) and (iii) terminates in a random voxel (red) within the STN. An example population of (E) 100 internal capsule fibers of passage and (F) 100 hyperdirect pathway axons. The inset in (F) shows that each hyperdirect pathway axon is comprised of a corticofugal axon with a branching collateral that terminates within the STN, whereas the inset in (E) shows that the internal capsule fibers of passage do not have a collateral.

We constructed multi-compartment cable models of myelinated axons to represent the hyperdirect pathway, as well as internal capsule fibers of passage, in NEURON (Fig 4). Both pathways consisted of a corticofugal axon passing through the internal capsule. The models representing the hyperdirect pathway where unique in that they had an axon collateral that branched from the corticofugal axon and terminated in the STN [20,21].

We used probabilistic tractography to define the trajectory of each corticofugal axon (Fig 4B). FSL’s probabilistic tractography tool (probtrackx) generated trajectories, or ‘streamlines’, which originated in the seed mask and terminated in the target masks (S3 Fig and Section I in S1 Text). Of the 13,219 corticofugal streamlines that were reconstructed with probabilistic tractography, we randomly sampled 2,000 for use in our models. One thousand streamlines were used to model the internal capsule fibers of passage, and the other 1,000 streamlines were designated to the hyperdirect pathway. We fit a smoothing spline to each tractography-generated streamline to ensure a smooth trajectory for each streamline (Fig 4C and S4 Fig).

For the hyperdirect pathway axons, we modeled the collateral as a branch at a randomly chosen node of Ranvier along the corticofugal axon that was within the axial bounds of the STN (Fig 4D). A random voxel within the STN was selected as the termination point of the collateral. We then generated an arc connecting the branch point node of Ranvier and the termination point within the STN to define the collateral trajectory. If the collateral passed through the DBS electrode, we randomly selected a different voxel within the STN and recalculated the corresponding arc.

The geometric and electrical parameters of the corticofugal axons were defined from previously established models [37]. The myelinated axon was modeled with a double cable structure and the nodes of Ranvier contained active (i.e. voltage-gated fast Na^+^, persistent Na^+^, and slow K^+^ ion channel conductances) and passive (i.e. leak conductance, capacitance) membrane properties. The axon model compartments of the corticofugal axons were defined with a myelin diameter of 5.7 μm and the hyperdirect collaterals were defined with a myelin diameter of 1.8 μm. We divided each corticofugal axon into compartments (node of Ranvier, MYSA, FLUT, STIN) and calculated the coordinates of each compartment along the arc length of the streamline. The coordinates of each compartment for the hyperdirect collateral were defined in the same manner as the corticofugal axon. We shortened the collateral’s first node of Ranvier to 0.5 μm and shortened the distal end of the collateral so that it ended with a node of Ranvier. The terminal node was assigned passive membrane properties to minimize any role as a hyperexcitable locus for action potential initiation [38].

### 2.7. Axon model stimulation

The response of each individual axon model to the spatially- and temporally-varying DBS voltage distribution, Φ(x,y,z,t), was calculated with NEURON (S1 Video and S2 Video) [39]. For each pulse width (20–120 μs), with contact 2 set as the cathode and the IPG case set as the anode, we used a binary search algorithm to determine the stimulus amplitude, A, that was sufficient for generating propagating action potentials. The threshold stimulus amplitude was calculated to within 0.01 V. The axons were stimulated with 3 pulses and the criteria for activation was that the distal active nodes of Ranvier on the corticofugal axon had to generate a 1-to-1 response to each stimulus pulse. For both the internal capsule fibers of passage and hyperdirect pathway axons, we excluded axons from subsequent analyses that had thresholds greater than or equal to 150 V or initiated action potentials in the distal active nodes of Ranvier on the corticofugal axon. This resulted in 989 internal capsule fibers of passage and 1000 hyperdirect pathway axons. Subsequently, each of these axons for a given pathway were clustered randomly into 100 populations of 1000 axons in a bootstrapping manner (with replacement), to quantify the effects of variability in the distribution of the axon trajectories. The average and standard deviation of the number of activated axons for the 100 populations in response to a specific stimulation amplitude are presented.

We systematically changed several simulation parameters to ensure that the results converged on an accurate solution. The differences in stimulation threshold amplitudes for axons of the internal capsule fibers of passage were calculated. Two different analyses were performed: 1) we increased the mesh resolution in COMSOL from 1,429,416 to 2,347,048 tetrahedral elements; and 2) we decreased the time step in NEURON from 1 μs to 0.5 μs. Each of these changes resulted in less than 1.2% differences in the stimulation thresholds.

## 3. Results

PAMs are the integrated processing of imaging data from DBS patients with tractography and electrical stimulation modeling to provide a theoretical estimate of axonal pathway activation. In this study, we generated an example PAM using high-field (7T) MRI data to construct the patient model [40]. These images have higher signal-to-noise, voxel resolution, and contrast than the 1.5T or 3T MRIs typically collected for clinical DBS procedures [41].

**Fig 5:**
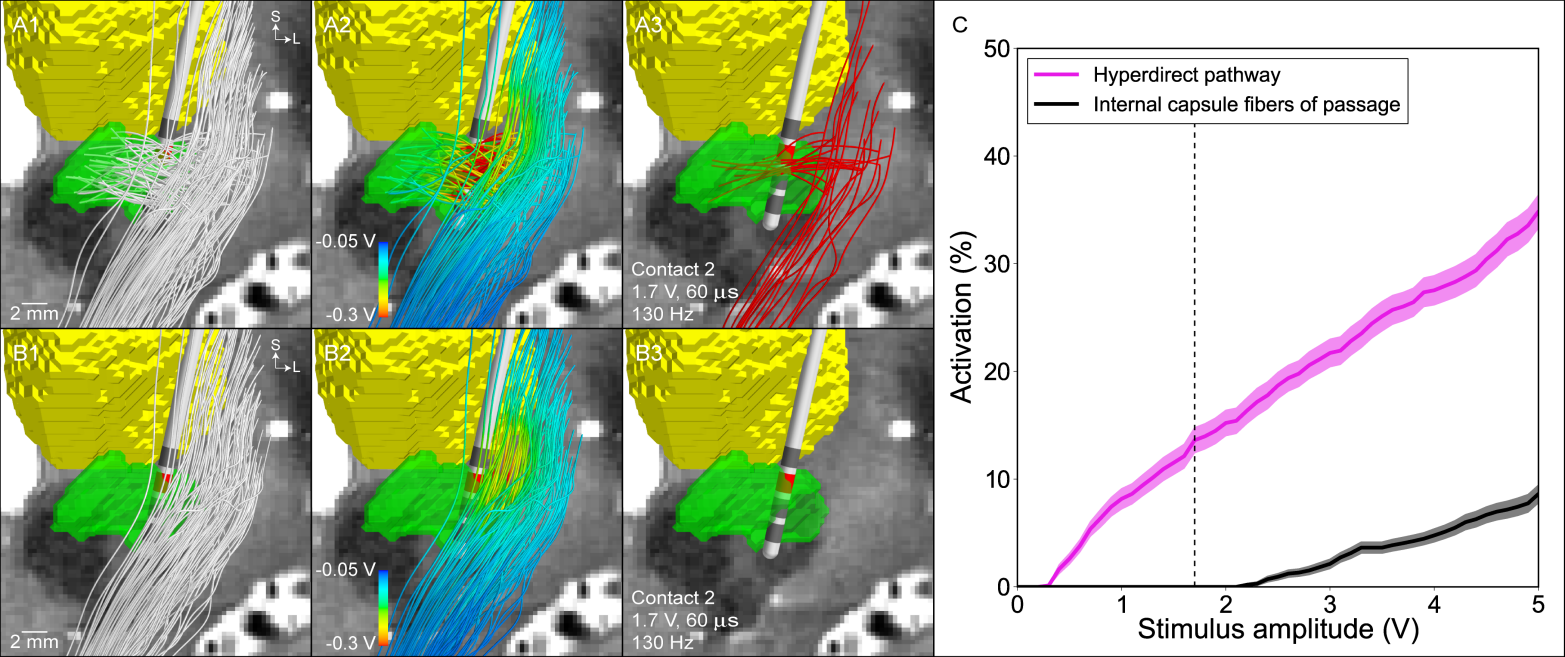
Model predictions for the activation of the hyperdirect pathway and internal capsule fibers of passage. Representative population of (A1) 100 hyperdirect pathway axons and (B1) 100 internal capsule fibers of passage (subthalamic nucleus–green, thalamus–yellow). (A2), (B2) The voltage distribution generated by -1.7 V applied at contact 2 is interpolated along the streamlines. (A3), (B3) The voltage distribution is used to stimulate the axon models, and those axons that are activated by the clinically effective stimulation setting (-1.7 V, 60 μs, 130 Hz) are shown in red. (C) Percent activation of each pathway as a function of the stimulation amplitude (contact 2 [cathode], IPG case [anode], 60 μs, 130 Hz). The dashed vertical line is the clinically effective stimulation amplitude.

We designed the patient-specific PAM to enable comparison of the DBS-induced activation of two sets of corticofugal axonal pathways. One set represented the hyperdirect pathway and the other represented the internal capsule fibers of passage. The activation of both pathways was calculated as a function of stimulation amplitude (Fig 5). At the clinically effective stimulation setting (contact 2 [cathode], IPG case [anode], 1.7 V, 60 μs, 130 Hz), the model predicted 13.6 ± 1.2% activation of the hyperdirect pathway and 0 ± 0% activation of the internal capsule fibers of passage (S2 Video).

The model predictions corresponded well with the clinical hypothesis that the hyperdirect pathway is directly activated during therapeutic subthalamic DBS. The steep slope of the hyperdirect recruitment curve also supports the clinical hypothesis that the degree of hyperdirect pathway activation is proportional to the degree of therapeutic benefit [25]. However, hyperdirect pathway activation is constrained by stimulation spread into the internal capsule fibers of passage. Significant activation of these internal capsule fibers of passage is known to generate unwanted side effects [27]. Previous electromyography-based estimates for DBS-induced muscle contractions have suggested that side effects begin to occur at ~10% activation of the internal capsule fibers of passage [16].

**Fig 6:**
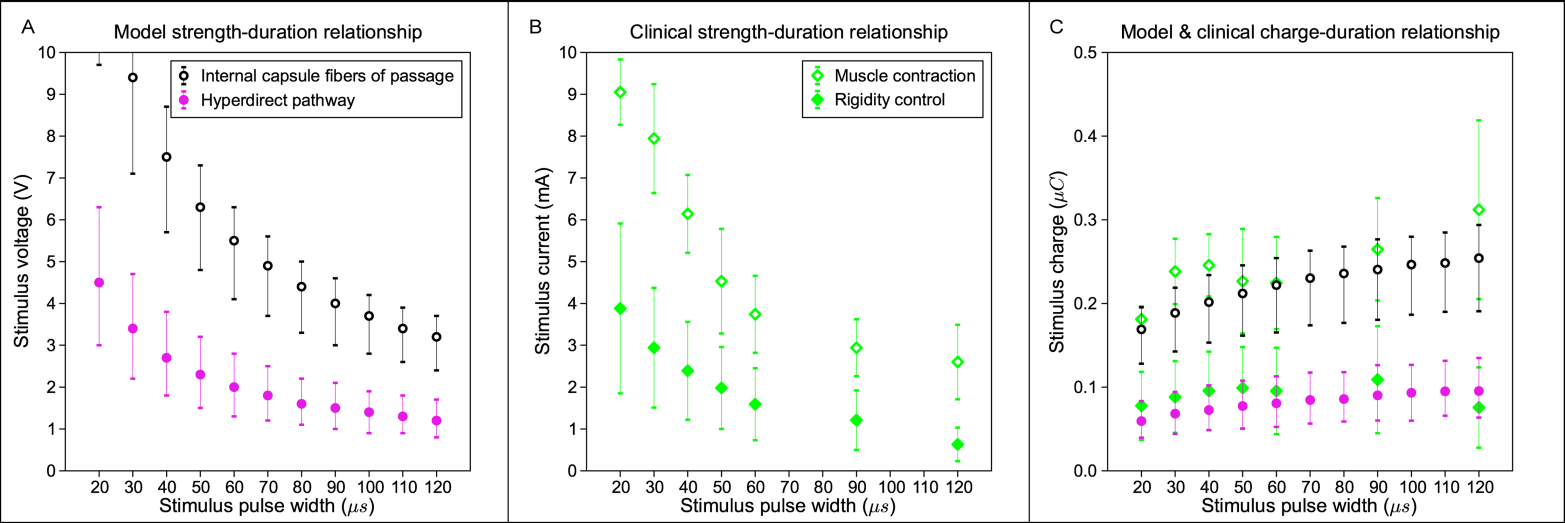
Model and clinical strength-duration and charge-duration curves. (A) Model threshold amplitudes for activation of the hyperdirect pathway (pink filled circle) and internal capsule fibers of passage (black open circle) at 15 ± 5% and 10 ± 5%, respectively. (B) Clinically-measured threshold amplitudes for DBS-induced rigidity control (green filled diamond) and muscle contractions (green open diamond) [42]. (C) Total charge injected during the cathodic phase of the stimulus for the threshold amplitudes shown in A and B.

A key concept in the clinical implementation of DBS is the “therapeutic window,” i.e. the stimulation amplitude range between the onset of therapeutic effects and the generation of side effects [28]. Typically, the electrode contact with the largest therapeutic window is the contact selected for chronic stimulation. Given that good therapeutic effects were generated in our example patient with ~15% activation of the hyperdirect pathway, we then quantified the stimulus amplitudes necessary to activate 15 ± 5% of the hyperdirect pathway as a function of the stimulus pulse width; thereby creating a hyperdirect strength-duration curve (Fig 6A). A similar internal capsule fibers of passage strength-duration curve was also generated for 10 ± 5% activation.

The results show that the amplitude window between direct activation of the hyperdirect pathway and the internal capsule fibers of passage increases with decreasing pulse width (Fig 6A). This theoretical calculation provides a possible biophysical explanation for the typical clinical practice of using short pulse widths to increase the therapeutic window [43]. In addition, our theoretical results, albeit from a single patient, match well with the strength-duration curves for clinically measured, DBS-induced rigidity control and muscle contractions (Fig 6B) [42]. To more directly compare the model and clinical strength-duration curves, which were generated with voltage-controlled and current-controlled IPGs, respectively, we plotted the results from Fig 6A and 6B as charge-duration curves (Fig 6C). The total charge injected during the cathodic phase of the stimulus was calculated with trapezoidal numerical integration for the stimulus amplitudes in Fig 6A and 6B. Of particular note was the tight congruence of the theoretical hyperdirect activation with clinical measurements on the control of rigidity (Fig 6C).

## 4. Discussion

This manuscript provides a detailed description of the technical steps to construct a patient-specific PAM. PAMs represent a new scientific tool for integrating brain mapping connectomics with the computational neuroscience of electrical stimulation modeling. An obvious application of PAMs is in the field of clinical DBS, where the concepts of pathway-targeted neuromodulation for the control of specific symptoms are currently under intense clinical investigation.

### 4.1. Next generation models of DBS

Over the last two decades, the clinical applications of DBS have evolved from a focus on movement disorders to expanded opportunities in treating psychiatric disorders and epilepsy. A common feature that potentially links these various disorders are the existence of dysfunctional brain circuit oscillations that can be overridden by direct extracellular stimulation of axonal pathways [1]. In turn, the application of DBS to brain circuit modulation presents an exciting opportunity to leverage the massive scientific efforts currently underway to map the human connectome [44,45]. However, most connectome-type projects rely on data derived from healthy subjects, whereas DBS is implemented in patients with neurological disorders, who have putative differences in their brain anatomy and axonal connections. In addition, we propose that an important aspect of integrating tractography with DBS modeling is to define methods that accurately predict the biophysical response of specific axonal pathways to electrical stimulation.

A key component of PAMs are the use of multi-compartment cable axon models to quantify the neural response to DBS. This is in contrast to more simplistic approaches to estimate brain regions where DBS-induced action potentials are likely to occur via activation volume predictor functions [46]. Only PAMs explicitly represent the transmembrane currents generated by extracellular stimulation, which are responsible for inducing membrane depolarization in the neural compartments closest to the active cathodic electrode contact [38]. These stimulation-induced inward currents open sodium channels, and if the polarization is sufficiently strong, an action potential will be generated. However, a wide range of factors dictate DBS-induced action potential generation including: 1) the electrode configuration, 2) the shape, duration, and frequency of the applied stimuli, 3) the electrical conduction properties of the brain tissue medium, 4) the geometry and trajectory of the axons, and 5) the membrane biophysics of the axons. In our experience, the most anatomically and electrically accurate method currently available to account for those various factors is a PAM.

In addition to PAMs, activation volume tractography (AVT) represents an alternative method to link tractography and stimulation. New academic software tools such as DBSproc [47] and Lead-DBS [48] facilitate the creation of AVT models. In general, both PAMs and AVT use similar methods to construct a patient-specific model of the anatomy and the DBS electrode location. The major differences reside in the methodology for predicting axonal pathway activation. AVT defines an activation volume around the DBS electrode contact and then uses the voxels contained within that activation volume as seeds for tractography. AVT can help identify pathways of interest in a DBS therapy, but is prone to generating erroneous results (e.g. anatomically nonexistent pathways) [49]. Alternatively, PAMs use tractography to define known anatomical pathways of interest a priori, and then calculates the biophysical response of those pathways to electrical stimulation. However, relative to AVT, PAMs are more difficult to develop and analyze. We propose that each method has its own merits and value, with the major comparison being speed and simplicity for AVT versus anatomical detail and biophysical realism for PAMs.

### 4.2. DBS modeling in clinical research

While connectome-based DBS modeling is still in its infancy, the applications for clinical investigation have already been numerous. DBS for depression represents one of the most active areas of investigation, with studies addressing the potential pathways directly activated by DBS [17,18,50], differences in pathway activation between alternative surgical targets [14], prospective identification of novel surgical targets [51], and probabilistic identification of pathways related to therapeutic benefit [4]. Similarly, wide-ranging efforts are currently underway in movement disorders, with numerous recent examples focused on the development of correlations between stimulation of various pathways and the control of tremor [12,13,52,53].

The results of this study provide theoretical insight into stimulation of the hyperdirect pathway during subthalamic DBS. Activation of the hyperdirect pathway has been hypothesized to be related to improvements in rigidity [54]. Our patient-specific biophysical branching model of hyperdirect collaterals in the STN provided an opportunity to more directly address that hypothesis (Fig 6), which necessitated a more anatomically realistic model than previous attempts to reconstruct the hyperdirect pathway [26,55–57]. This is because both the complex axonal trajectory and branching impact the activation threshold from extracellular stimulation [38]. The model results demonstrate robust activation of the hyperdirect pathway at the clinical stimulation setting in our example patient (Fig 5). We also observed strong congruence between strength-duration curves for activation of the hyperdirect pathway in our model and population averages of clinically-measured rigidity control from DBS (Fig 6). These results support the concept that future PAM analyses, applied to a population of DBS patients, may help in identifying correlations between direct activation of a particular pathway and modulation of a clinical symptom.

### 4.3. Study limitations and future work

The PAM created for this study represents a highly detailed patient-specific DBS computational model. However, as with any model, multiple limitations and caveats exist. PAMs are able to predict the direct activation of individual axonal pathways to a stimulus pulse, but it should be noted that PAMs do not quantify the network-level modulatory effects of DBS. However, such questions may eventually be addressed by the future combination of PAMs with large-scale network activity models [58].

Image registration and definition of the DBS electrode location in the brain represent some of the most important sources of error in creating patient-specific DBS models. We used established registration algorithms to transform the 7T images, 1.5T image, and CT image to a common coordinate system [41], and registration quality was verified using visual inspection to ensure that the subcortical and cortical boundaries aligned. In addition, we used established methods to minimize error in alignment of a model DBS electrode to the electrode artifact in the CT [59].

Once the image pre-processing is complete, the patient-specific FEM and tractography-based axon models can be integrated together. While DBS FEMs are only an approximation of a highly complex phenomenon [60], they are able to match *in vivo* experimental recordings of the voltage distribution in the brain with impressive fidelity [30]. However, our latest advances in DBS FEM parameterization reinforce the importance of incorporating all of the electrical details described in our PAM workflow to generate the most accurate results [31,32].

The multi-compartment cable models of axons we used were stylized to a single diameter and ignore some of the complex branching patterns of real axons [20,21]. These biophysical limitations are also coupled to the general limitations of tractography, which are well documented elsewhere [61] and are directly applicable to its use in PAMs. Nonetheless, tractography does represent the only non-invasive method to reconstruct structural connectivity on a patient-specific basis [44].

One area of necessary future development is refinement to the pathway reconstruction techniques and biophysical axon models. In the case of the hyperdirect pathway, as the collaterals terminate near the active DBS electrode contacts, consideration should be taken regarding the termination points of the streamlines. We initially attempted to use tractography to reconstruct the hyperdirect terminations within the STN [55,56,62]; however, the reconstructions through the grey matter were very tortuous and anatomically unrealistic. Anatomical tracing studies have shown that the hyperdirect pathway often branches upon entering the posterio-dorso-lateral aspect of the STN and collaterals terminate throughout the STN [19–21,63]. Additionally, studies have shown that the hyperdirect collaterals are typically less than 1 μm in diameter [20,64]. And in the human internal capsule, there is a wide range of axon diameters from <1–10 μm [65,66]. Each of these anatomical details will affect the predictive power of the model and represent opportunities for future improvement.

## 5. Conclusions

PAMs represent advanced computational tools with potential to augment clinical investigations on the mechanisms of DBS. The functional goal of PAMs is to provide quantitative patient-specific predictions on the axonal pathways directly activated by DBS, and then enable linkage of those pathway activation metrics to clinical outcome measures associated with specific symptoms. In addition, PAMs could one day be coupled with functional neuroimaging to help investigate the network-level neuromodulatory effects of DBS [67,68].

## Supporting information

S1 Fig Equivalent electrical circuit diagram of the implanted DBS system for voltage-regulated, monopolar stimulation. The circuit included representations of the blocking capacitors (C_Block_), extension wire resistance (R_Extension_), lead wire resistance (R_Lead_), electrode-tissue interface with a double-layer capacitance (C_dl_) and Faradaic resistance (R_Faradaic_) in parallel, and tissue resistance (R_Tissue_). A ‘parasitic’ capacitance (C_Parasitic_) and ‘parasitic’ resistance (R_Parasitic_) were included in parallel with the load of the DBS system. (A) During the cathodic phase the circuit is driven by the voltage source (V_Applied_) (60 μs). (B) During the first portion of the interphase interval the voltage source is disconnected from the circuit (10 μs), and (C) during the second portion of the interphase interval the parasitic capacitance and parasitic resistance are also disconnected (70 μs). (D) During the passive charge recovery phase the DBS system load is connected to ground, and the parasitic capacitance and parasitic resistance are connected to each other (3.686 ms).
(TIF)

S2 Fig Patient-specific definition of the encapsulation layer conductivity. The impedance of the finite element model (FEM) (‘Static’, black dashed line) and implanted DBS system model (‘Waveform’, black solid line) as a function of the encapsulation layer conductivity for contact 2. To replicate the Medtronic clinical impedance measurement (crosshair), we calculated the implanted DBS system model impedance at 70 μs into an 80 μs pulse. The difference between the clinical impedance measured with the Medtronic programming device and the two model impedances is shown in purple.
(TIF)

S3 Fig Seed and target masks used by the probabilistic tractography algorithm to generate streamlines representing corticofugal axons. The subcortical nuclei outlined on the (A) T1-weighted image and (B) T2-weighted coronal image (subthalamic nucleus [STN]—green, substantia nigra–orange, red nucleus–red, thalamus–yellow, putamen–purple, globus pallidus externus–light blue, globus pallidus internus–dark blue). The 3 pink lines indicate the seed and target masks shown in A2-A4 and B2-B4. (A2), (B2) The seed mask was defined as the white matter between the thalamus and lenticular nucleus, 1.2 mm superior to the STN. (A3), (B3) The superior target mask was defined as the white matter between the thalamus and lenticular nucleus, 10.8 mm superior to the seed mask. (A4), (B4) The inferior target mask was defined as the cerebral peduncle of the midbrain, 17.2 mm inferior to the seed mask.
(TIF)

S4 Fig Differences between a tractography-generated streamline and a smoothing spline fit to a tractography-generated streamline. (A-C) Based off of corticofugal streamline shown in Fig 4C. (A) Extracellular voltage at the axon compartment midpoints along the tractography-generated streamline (blue) and spline-based streamline (black). (B) Extracellular voltage at the nodal compartment midpoints along the tractography-generated streamline and spline-based streamline. (C) Second nodal differences of the extracellular voltages along the tractography-generated streamline and spline-based streamline. (D) Stimulus threshold errors and (E) recruitment curves for the internal capsule fibers of passage axon models defined from the tractography-generated streamlines and spline-based streamlines.
(TIF)

S1 Table Imaging parameters. All MRI scans were acquired pre-operatively while the CT scan was acquired post-operatively. T2W and SW images were acquired in both coronal and axial orientations.
(PDF)

S2 Table Software programs utilized in the scientific workflow. (PDF)

S3 Table Isotropic conductivities for tissue types. (PDF)

S4 Table Images and methods for segmenting structures. (PDF)

S1 Text Supplementary methods. (PDF)

S1 Video Hyperdirect pathway axon model response to stimulation. In this example, the stimulation setting chosen is suprathreshold and thus generates an action potential that propagates orthodromically and antidromically (subthalamic nucleus–green). All four images are simultaneously changing over time to show: (A) the extracellular voltage distribution generated by the DBS electrode that is used to stimulate the model axon; and (B) the change in transmembrane voltage in response to stimulation. The line plots show the change in voltage at the node of Ranvier where action potential initiation occurs (black arrow).
(MP4)

S2 Video Model predictions for the response of 100 hyperdirect pathway axons and 100 internal capsule fibers of passage to the clinically effective stimulation setting. All five images are simultaneously changing over time to show (subthalamic nucleus–green; thalamus–yellow): (Left) the extracellular voltage distribution generated by contact 2 (red) that is used to stimulate the model axons; (Inset) the time course of the stimulus waveform; and (Right) the membrane voltage response to stimulation. For this stimulation setting (contact 2 [cathode], IPG case [anode], 1.7 V, 60 μs, 130 Hz), 14 hyperdirect pathway axons and zero internal capsule fibers of passage generate propagating action potentials in response to each stimulus pulse.
(MP4)

## Abbreviations

AVT: activation volume tractography
CSF: cerebrospinal fluid
CT: computed tomography
DBS: deep brain stimulation
DW: diffusion-weighted
ETI: electrode-tissue interface
FEM: finite element model
FA: fractional anisotropy
IPG: implantable pulse generator
MRI: magnetic resonance image
PD: Parkinson’s disease
PAM: pathway-activation model
STN: subthalamic nucleus
SW: susceptibility-weighted
T1W: T1-weighted
T2W: T2-weighted
