# Supplementary figures and images for: Creating and parameterizing patient-specific deep brain stimulation pathway-activation models using the hyperdirect pathway as an example
Source: PLoS One. 2017 Apr 25;12(4):e0176132. doi: 10.1371/journal.pone.0176132 (PMC5404874; doi:10.1371/journal.pone.0176132)

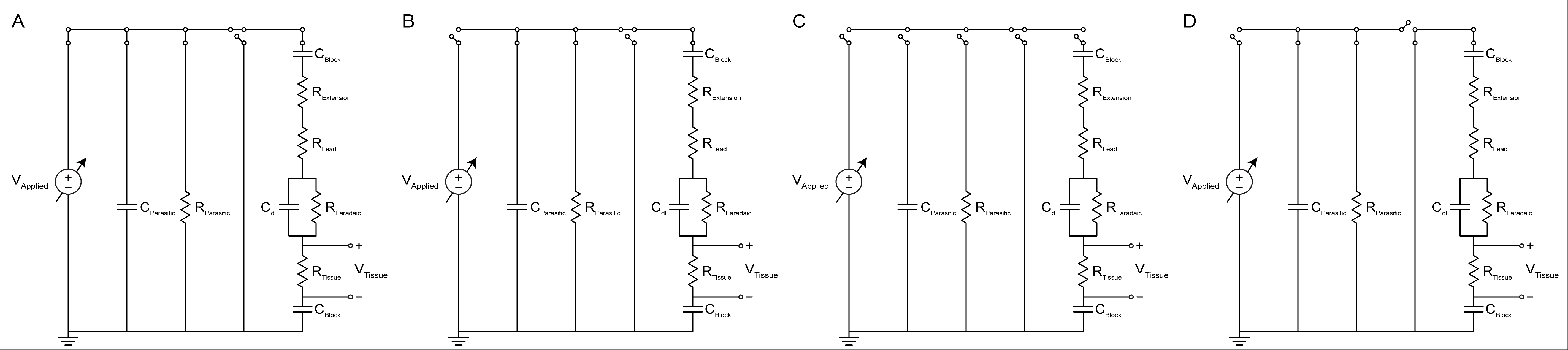

Supplement: S1 Fig — The circuit included representations of the blocking capacitors (CBlock), extension wire resistance (RExtension), lead wire resistance (RLead), electrode-tissue interface with a double-layer capacitance (Cdl) and Faradaic resistance (RFaradaic) in parallel, and tissue resistance (RTissue). A ‘parasitic’ capacitance (CParasitic) and ‘parasitic’ resistance (RParasitic) were included in parallel with the load of the DBS system. (A) During the cathodic phase the circuit is driven by the voltage source (VApplied) (60 μs). (B) During the first portion of the interphase interval the voltage source is disconnected from the circuit (10 μs), and (C) during the second portion of the interphase interval the parasitic capacitance and parasitic resistance are also disconnected (70 μs). (D) During the passive charge recovery phase the DBS system load is connected to ground, and the parasitic capacitance and parasitic resistance are connected to each other (3.686 ms). (TIF) [file pone.0176132.s001.tif]

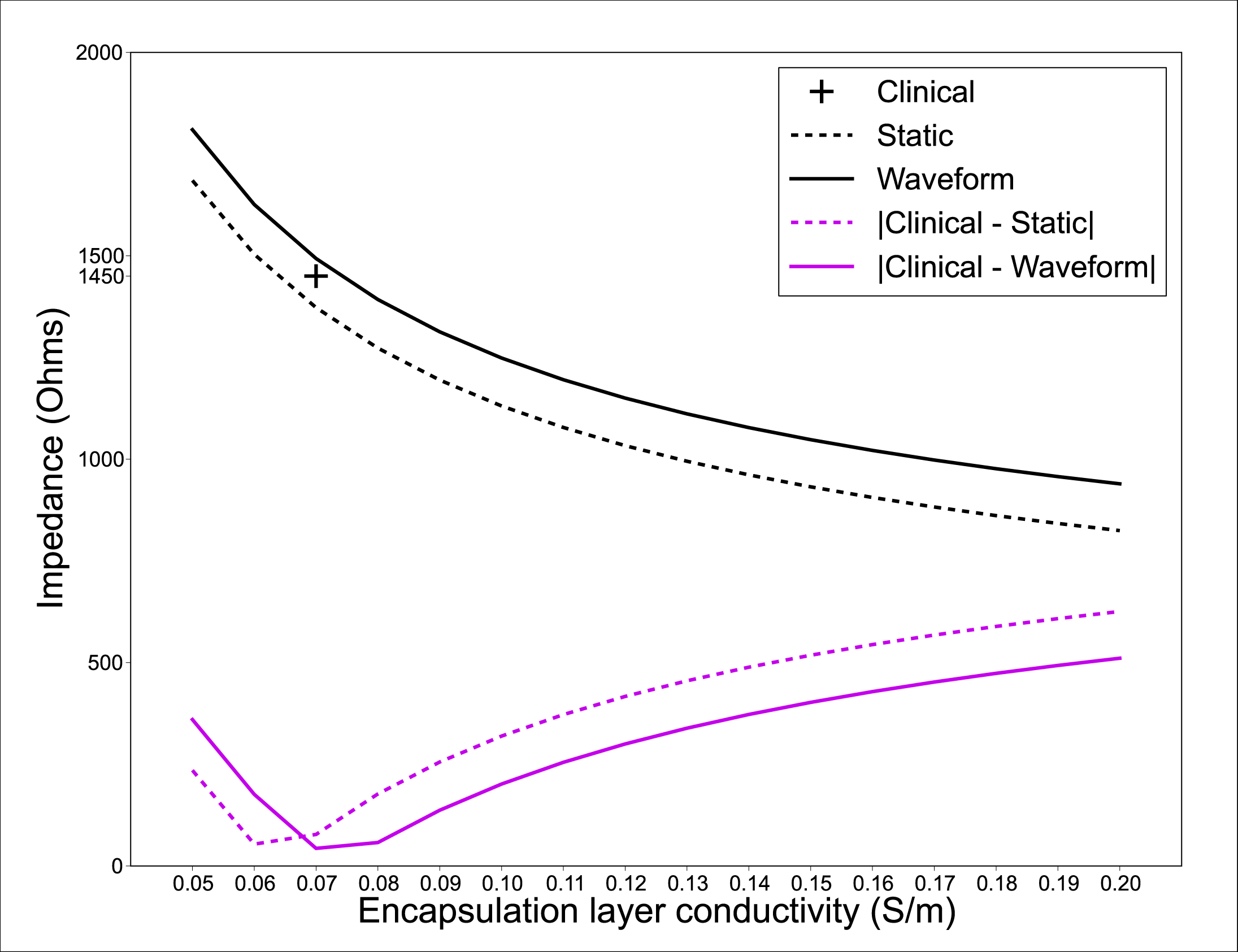

Supplement: S2 Fig — The impedance of the finite element model (FEM) (‘Static’, black dashed line) and implanted DBS system model (‘Waveform’, black solid line) as a function of the encapsulation layer conductivity for contact 2. To replicate the Medtronic clinical impedance measurement (crosshair), we calculated the implanted DBS system model impedance at 70 μs into an 80 μs pulse. The difference between the clinical impedance measured with the Medtronic programming device and the two model impedances is shown in purple. (TIF) [file pone.0176132.s002.tif]

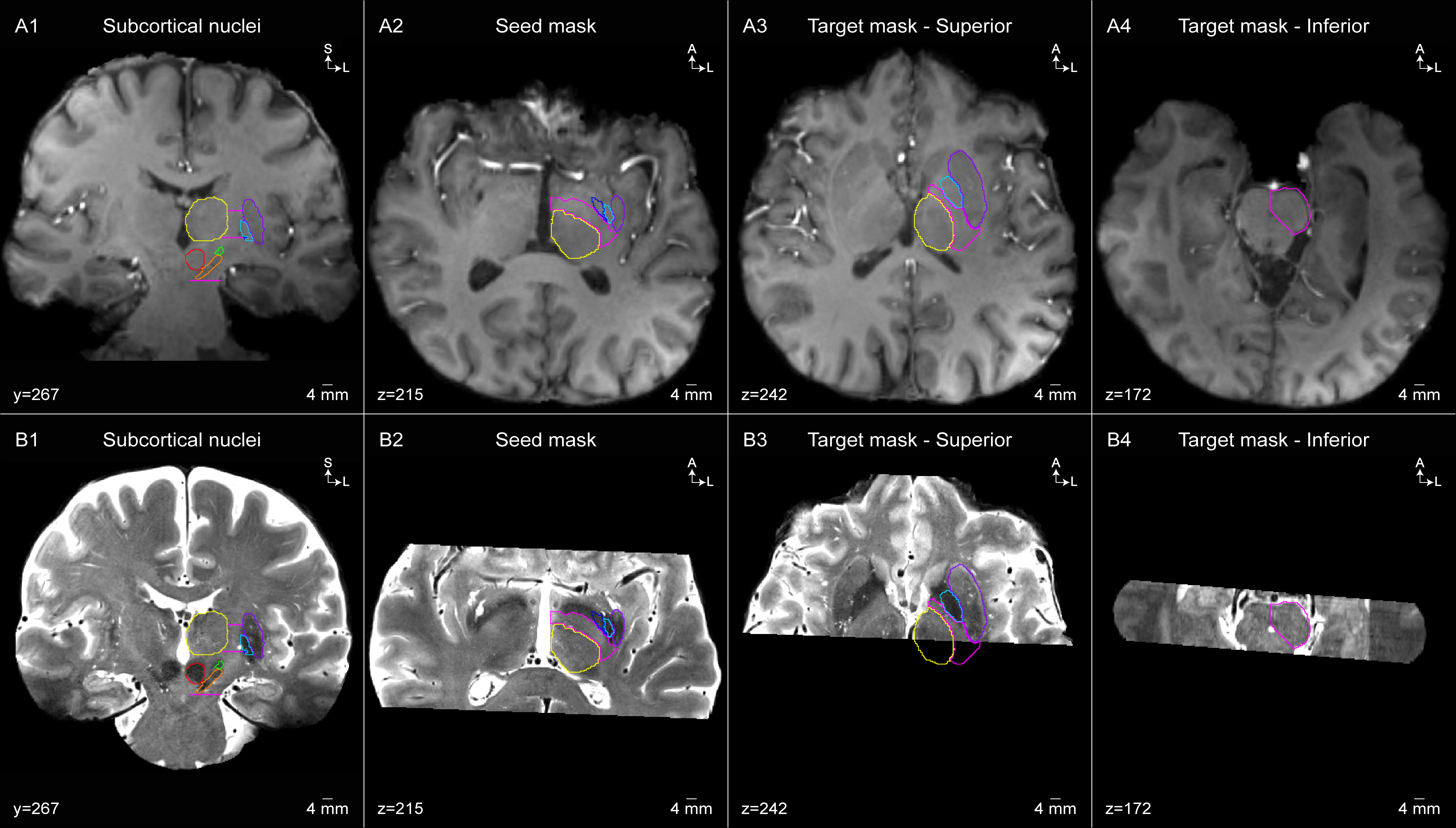

Supplement: S3 Fig — The subcortical nuclei outlined on the (A) T1-weighted image and (B) T2-weighted coronal image (subthalamic nucleus [STN]—green, substantia nigra–orange, red nucleus–red, thalamus–yellow, putamen–purple, globus pallidus externus–light blue, globus pallidus internus–dark blue). The 3 pink lines indicate the seed and target masks shown in A2-A4 and B2-B4. (A2), (B2) The seed mask was defined as the white matter between the thalamus and lenticular nucleus, 1.2 mm superior to the STN. (A3), (B3) The superior target mask was defined as the white matter between the thalamus and lenticular nucleus, 10.8 mm superior to the seed mask. (A4), (B4) The inferior target mask was defined as the cerebral peduncle of the midbrain, 17.2 mm inferior to the seed mask. (TIF) [file pone.0176132.s003.tif]

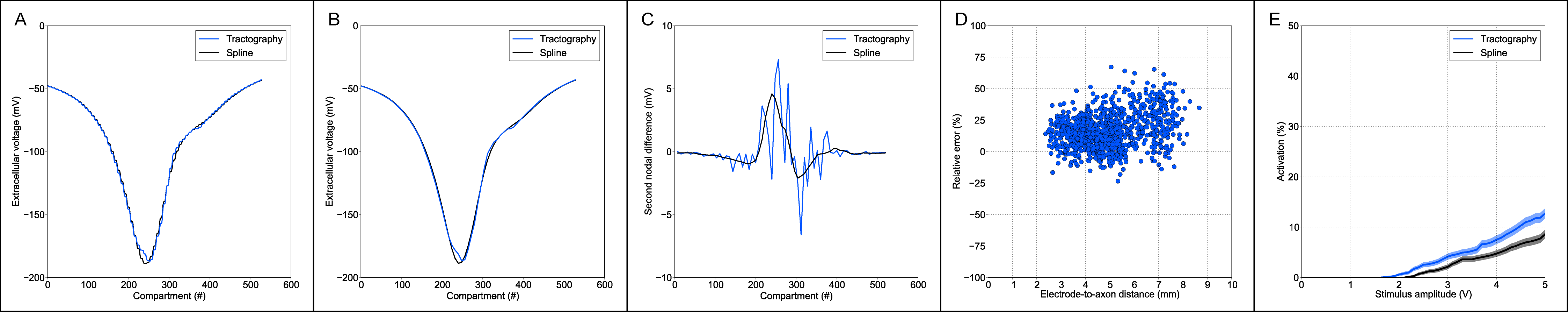

Supplement: S4 Fig — (A-C) Based off of corticofugal streamline shown in Fig 4C. (A) Extracellular voltage at the axon compartment midpoints along the tractography-generated streamline (blue) and spline-based streamline (black). (B) Extracellular voltage at the nodal compartment midpoints along the tractography-generated streamline and spline-based streamline. (C) Second nodal differences of the extracellular voltages along the tractography-generated streamline and spline-based streamline. (D) Stimulus threshold errors and (E) recruitment curves for the internal capsule fibers of passage axon models defined from the tractography-generated streamlines and spline-based streamlines. (TIF) [file pone.0176132.s004.tif]
