# Supplementary material for: Creating and parameterizing patient-specific deep brain stimulation pathway-activation models using the hyperdirect pathway as an example
Source: PLoS One. 2017 Apr 25;12(4):e0176132. doi: 10.1371/journal.pone.0176132 (PMC5404874; doi:10.1371/journal.pone.0176132)
Supplement: S1 Table — All MRI scans were acquired pre-operatively while the CT scan was acquired post-operatively. T2W and SW images were acquired in both coronal and axial orientations. (PDF) [file pone.0176132.s005.pdf]

S1 Table. Imaging parameters. All MRI scans were acquired pre-operatively while the CT scan was acquired post-operatively. T2W and SW images were acquired in both coronal and axial orientations.

Image	Sequence	FOV (mm^3)	Matrix	Voxel resolution (mm^3)	Other parameters	Acquisition time (minutes)
1.5T T1W	3D gradient echo MPRAGE	250x187x176	256x192x176	0.98x0.98x1.0	TR = 1650 ms<br>TI = 1100 ms<br>TE = 3.02 ms<br>Flip angle = 15°	10
7T T1W	3D gradient echo MPRAGE	230x187x153	312x384x256	1.0x1.0x1.0	TR = 3100 ms<br>TI = 1500 ms<br>TE = 3.5 ms<br>Flip angle = 6°<br>Acceleration factor of 2 (GRAPPA) along the phase-encoding direction.	6.5
7T T2W	2D turbo spin echo	200x200x26	512x512x26	0.39x0.39x1.0	TR = 9000 ms<br>TE = 58 ms<br>Flip angle = 150°<br>Acceleration factor of 3 (GRAPPA) along the phase-encoding direction.	7.5
7T SW	3D flow-compensated gradient echo	200x200x48	512x512x60	0.39x0.39x0.8	TR = 28 ms<br>TE = 21 ms<br>Flip angle = 17°<br>Pixel bandwidth = 121 Hz/pixel<br>6/8 partial Fourier parallel imaging using an acceleration factor of 2 (GRAPPA) along the phase-encoding direction.	4
7T DW	Single refocused 2D single-shot spin echo echo planar imaging	204x204x99	136x136x66	1.5x1.5x1.5	TR = 4896 ms<br>TE = 56 ms<br>Flip angle = 90°<br>Pixel bandwidth = 1671 Hz/pixel<br>Acceleration factor of 3 (GRAPPA).	4.5
CT	-	302x200x302	512x334x512	0.59x0.6x0.59	120 kV<br>315 mAs<br>Gantry tilt = 0.0°	0.5
