# Supplementary material for: Creating and parameterizing patient-specific deep brain stimulation pathway-activation models using the hyperdirect pathway as an example
Source: PLoS One. 2017 Apr 25;12(4):e0176132. doi: 10.1371/journal.pone.0176132 (PMC5404874; doi:10.1371/journal.pone.0176132)
Supplement: S2 Table — (PDF) [file pone.0176132.s006.pdf]

S2 Table. Software programs utilized in the scientific workflow.

Software	Version	Website	Citation
COMSOL Multiphysics	5.1	comsol.com/comsol-multiphysics	-
Python	2.7.8	python.org	-
MATLAB	8.0.0.783	mathworks.com/products/matlab	-
NEURON	7.3	neuron.yale.edu	Hines and Carnevale, 2001
FSL	5.0.7	fsl.fmrib.ox.ac.uk/fsl/fslwiki	Jenkinson et al., 2012
Freesurfer	5.3.0	surfer.nmr.mgh.harvard.edu	Fischl, 2012
Seg3D	2.3.0	seg3d.org	-
Cicerone	-	ciceronedbs.org	Miocinovic et al., 2007
MeshLab	1.3.3	meshlab.sourceforge.net	-
3DSlicer	4.4.0	slicer.org	-
