# Supplementary material for: Creating and parameterizing patient-specific deep brain stimulation pathway-activation models using the hyperdirect pathway as an example
Source: PLoS One. 2017 Apr 25;12(4):e0176132. doi: 10.1371/journal.pone.0176132 (PMC5404874; doi:10.1371/journal.pone.0176132)
Supplement: S3 Table — (PDF) [file pone.0176132.s007.pdf]

**S3 Table**. Isotropic conductivities for tissue types.

| Tissue | Isotropic conductivity (S/m) |
|---|---|
| Grey matter | 0.23 |
| White matter | 0.14 |
| Cerebrospinal fluid | 1.45 |
| Glial scar | 0.07 |
| Muscle | 0.32116 |
| Tendon | 0.38271 |
| Bone | 0.020157 |
| Fat | 0.022405 |
| Skin | 0.00020006 |
| Disk | 0.65 |
| Blood | 0.7 |
| Air | 1e-12 |
