# Supplementary material for: Creating and parameterizing patient-specific deep brain stimulation pathway-activation models using the hyperdirect pathway as an example
Source: PLoS One. 2017 Apr 25;12(4):e0176132. doi: 10.1371/journal.pone.0176132 (PMC5404874; doi:10.1371/journal.pone.0176132)
Supplement: S4 Table — (PDF) [file pone.0176132.s008.pdf]

S4 Table. Images and methods for segmenting structures.

Structure	MRI	Method
Thalamus	1.5T T1W	Manual fitting
Caudate	7T T1W	Manual segmentation
Putamen	7T T2W (or SW) axial	Manual segmentation
Globus pallidus externus	7T T2W (or SW) axial	Manual segmentation
Globus pallidus internus	7T T2W (or SW) axial	Manual segmentation
Subthalamic nucleus	7T T2W (or SW) coronal	Manual segmentation
Substantia nigra	7T T2W (or SW) coronal	Manual segmentation
Red nucleus	7T T2W (or SW) coronal	Manual segmentation
Contralateral hemisphere	1.5T T1W	Automated segmentation
Cerebrospinal fluid	1.5T T1W	Automated segmentation
