# Supplementary material for: Creating and parameterizing patient-specific deep brain stimulation pathway-activation models using the hyperdirect pathway as an example
Source: PLoS One. 2017 Apr 25;12(4):e0176132. doi: 10.1371/journal.pone.0176132 (PMC5404874; doi:10.1371/journal.pone.0176132)
Supplement: S1 Text — (PDF) [file pone.0176132.s009.pdf]

Creating and parameterizing patient-specific deep brain stimulation pathway-activation models using the hyperdirect pathway as an example

Kabilar Gunalan, Ashutosh Chaturvedi, Bryan Howell, Yuval Duchin, Scott F. Lempka, Remi Patriat, Guillermo Sapiro, Noam Harel, Cameron C. McIntyre

---

S1 Text. Supplementary methods.

A. Software programs

We used several software programs in the model development (S2 Table). Although each program has methods for data visualization, we wrote custom Python scripts using open source software libraries such as Mayavi (docs.enthought.com/mayavi/mayavi), to combine all data into a single visualization environment. Some of these scripts are available at the McIntyre Lab GitHub site (https://github.com/mcintyrelab).

B. Image scanning parameters

We scanned the subject using an actively shielded 7T magnet, using SC72 gradients capable of 70 mT/m and a 200 T/m/s slew rate, driven by a Siemens console (Erlangen, Germany). We acquired all 7T images with a 32-element head array coil (Nova Medical, Inc., Burlington, MA) and with the MRI vendor's 3D distortion correction, which compensates for geometrical distortions originating from gradient nonlinearities. We acquired T2W and SW images in both the coronal and axial orientations centered around the basal ganglia. We acquired DW images with diffusion gradients applied along 50 uniformly distributed directions (b-value = ~1500 s/mm^2). We also acquired four additional non-DW images (b-value = 5 s/mm^2). We repeated the diffusion acquisition with the same parameters and head position but with the opposite phase encoding direction to allow for distortion correction.

C. Image pre-processing and co-registration

We corrected DW images for distortions from eddy currents using FSL's `eddy` tool and from magnetic field inhomogeneities using FSL's `topup` tool. We registered all images to the same coordinate system using Advanced Normalization Tools (ANTs) or FSL's linear image registration tool (`flirt`). To facilitate the registration, we extracted non-brain structures from the 1.5T T1W, 7T T1W, 7T T2W, 7T SW, and 7T DW b_0 images using FSL's brain extraction tool (`bet`). As the T2W and SW images have in-plane resolutions of 0.39 mm, the common coordinate system had an isotropic resolution of 0.4 mm.

We used a post-operative CT image to verify the final location of the implanted DBS electrode. First, we registered the CT image to the pre-operative T1W image and then in Cicerone we positioned a model Medtronic 3389 DBS electrode to match the electrode artifact in oblique slices (Fig 2C) (Hemm et al., 2009). We exported the coordinates of the collinear contact centers from Cicerone.

D. Imaging space

In Python, MATLAB, and COMSOL, we positioned all objects according to flipped, scaled voxel space (x,y,z) (Equation S1). First, we scaled each coordinate in voxel space (i,j,k) by the voxel dimensions ($voxel_x$,$voxel_y$,$voxel_z$), thereby converting the coordinates to millimeters. Next, we obtained the x-axis orientation from the sign of the determinant of the T1W image qform matrix. We used FSL's `fslorient` tool with the `-getqform` flag to obtain the qform matrix. This process can be summarized as follows, where $\circ$ is the Hadamard product:

$$\begin{pmatrix} x \\ y \\ z \end{pmatrix} = \begin{pmatrix} i \\ j \\ k \end{pmatrix} \circ \begin{pmatrix} voxel_x \\ voxel_y \\ voxel_z \end{pmatrix} \circ \begin{pmatrix} sign(|\mathrm{qform}|) \\ 1 \\ 1 \end{pmatrix} \tag{S1}$$

Although true anatomical space, as defined by the qform of the NIfTI header, can also be rotated, translated, and sheared, we have elected to ignore those transforms since Python and MATLAB do not handle those transformations easily.

E. Conductivity tensor field construction

Howell and McIntyre (2017) showed that the heterogeneity in the soft tissues of the head effect the voltage distribution generated by DBS. They defined conductivities for each region outside of the brain instead of a lumped equivalent value for all regions, and compared the results. However, specification of these regions on a patient-specific basis is difficult as it requires manual segmentation of a MRI. Thus we have opted to transform the soft tissues from the multimodal imaging-based detailed anatomical (MIDA) model of the human head and neck (Iacono et al., 2015). We used a variant of the MIDA model, specifically the $MIDA_{12}$ model as described by Howell and McIntyre (2017). The following is a list of steps for transforming the $MIDA_{12}$ regions outside of the brain to the patient's T1W space (Fig 3A): 1) Segmented the patient brain-extracted 1.5T T1W image into tissue types (grey matter, white matter, cerebrospinal fluid [CSF]) with FSL's `fast` tool. 2) Registered the $MIDA_{12}$ brain (grey matter, white matter, and CSF) to the tissue-type segmented patient brain from step 1 with FLIRT using 12 degrees of freedom. 3) Transformed $MIDA_{12}$ head segmentations to the patient T1W space with nearest neighbor interpolation using the transformation from step 2. The following steps will refer to the $MIDA_{12}$ mask that is now in patient space. 4) Calculated overlap in the $MIDA_{12}$ spinal cord/brainstem regions and patient brain. Subtracted remaining spinal cord/brainstem regions from $MIDA_{12}$ mask. 5) Subtracted the patient's brain region from $MIDA_{12}$ mask, thereby leaving soft tissues, skull, and residual brain regions from $MIDA_{12}$. 6) Removed the residual brain regions from $MIDA_{12}$, specifically the dura, grey matter, white matter, and CSF. Reassigned this region to CSF. 7) Added back the remaining $MIDA_{12}$ spinal cord/brainstem regions. 8) Added the tissue-type segmented patient brain from step 1.

The processing steps to transform the $MIDA_{12}$ segmentations to patient space preserve the anatomy of the patient's brain. However, the $MIDA_{12}$ skull is partially removed to ensure preservation of the patient's brain. Another limitation with this method is that the outer boundary of the patient's head is not preserved. Furthermore, anatomical variability exists from patient to patient and thus using the $MIDA_{12}$ segmentations to define regions outside of the brain is only an approximation.

Once this classification mask was created (Fig 3A), we then constructed conductivity tensors, S, that were anisotropic within the brain and isotropic in the encapsulation layer surrounding the electrode and outside of the brain (S3 Table). We constructed the anisotropic and inhomogeneous conductivity tensors within the brain by first using FSL's `dtifit` tool to estimate the diffusion tensors, D, from the DW images. We used FSL's `vecreg` tool to transform the diffusion tensors from DW to T1W space. We then used `vecreg` to down sample this image by a factor of 2. Using eigen decomposition, we decomposed these diffusion tensors to diffusion eigenvalues and diffusion eigenvectors. Using the preservation of tensor electrical load approach, we scaled the diffusion eigenvalues at each voxel to create conductivity eigenvalues (Howell and McIntyre, 2016). This scalar mapping was dependent on whether the tensor was within grey matter, white matter, or CSF. Finally, using the eigenvalues of S, we reconstructed S by assuming that D and S have the same eigenvectors (Basser et al., 1994). We saved a text file with the tensor values at each voxel, resulting in a matrix with a size of M x 9. The first three columns are the x, y, and z coordinates, and the last six columns are the upper triangular portion of S. We imported this text file into COMSOL and the conductivity tensors at each voxel location were interpolated onto the mesh nodes. We visualized the conductivity tensors in Python (Mayavi) to ensure correct registration with the T1W image and correct orientation of the tensors (Fig 3B/C/D). For this visualization, we used the conductivity eigenvalues to calculate the fractional anisotropy of each tensor.

We used the isotropic conductivity of the encapsulation layer to match the implanted DBS system model impedance to the clinically-measured impedance using the following steps (S2 Fig) (Butson et al., 2006): 1) We used the Medtronic programming device to measure the electrode impedance for contact 2 (-0.7 V, 80 μs, 100 Hz). 2) We solved the FEM, with contact 2 set as the working electrode, for a range of encapsulation layer conductivities (0.05 – 0.2 S/m) (Grill and Mortimer, 1994; Butson et al., 2006). For each encapsulation layer conductivity, we calculated the FEM impedance using Ohm's law (i.e. divided the electrode voltage applied by the total current produced at the electrode surface). 3) For contact 2, to replicate the impedance measurements of the Medtronic programming device, we calculated the implanted DBS system model impedance using Ohm's law by setting R_{Tissue} equal to the FEM impedance, applying a -0.7 V 80 μs stimulus to the circuit in S1 Fig, and measuring the current through R_{Tissue} at 70 μs. 4) We selected the encapsulation layer conductivity that minimized the absolute difference between the implanted DBS system model impedance calculated in Step 3 and the clinical impedance measured with the Medtronic programming device in Step 1, for contact 2 (i.e. 0.07 S/m) (S2 Fig). With the encapsulation layer conductivity set to 0.07 S/m, the implanted DBS system model impedance calculated in Step 3 with contact 2 set as the working electrode was 1493 Ω. It should be noted that impedance is a misnomer. Loads from the IPG are not measured at steady state with sinusoidal stimuli and thereby are not impedances. Nonetheless, dynamic loads measured with IPGs are referred to as impedances, so we chose to use the same terminology.

F. Surface mesh processing

To define the brain and head volumes, we used `bet` to extract the patient-specific inner skull surface mesh from the 1.5T T1W image (Fig 2A/B), and used Seg3D to extract the outer head surface mesh from the $MIDA_{12}$ volume (Section E in S1 Text). We ran `bet` with the fractional intensity threshold that yielded the best qualitative extraction of the inner skull surface. For the

patient presented in this manuscript, we used a fractional intensity value of 0.4. This tessellated surface mesh typically results in a high number of faces, which would cause COMSOL to take a long time to solve. Therefore, we imported this surface mesh into MeshLab and decimated to reduce the number of faces to less than 1000. Specifically, we applied the 'Quadratic Edge Collapse Decimation' filter three times with the percentage reduction set to 0.5. After each decimation step, we applied the 'Laplacian smooth (surface preserve)' filter to maintain a uniform distribution of faces. We registered the tissue-type segmented $MIDA_{12}$ brain to the tissue-type segmented patient brain with FLIRT using 12 degrees of freedom (Section E in S1 Text). We used this transformation to transform the $MIDA_{12}$ outer head surface mesh to the patient T1W space. In MeshLab, we converted these triangular meshes to quadratic meshes with the 'Tri to Quad by 4-8 subdivision' filter and exported it as an *.off file. To ensure there was no overlap, we imported the two meshes into MeshLab. Because COMSOL cannot import quadratic meshes, we converted these meshes to a non-uniform rational basis spline (NURBS) file format in MATLAB using the 'NURBS Toolbox by D.M. Spink'. We then imported these meshes into COMSOL to ensure there was no overlap.

G. Finite element model – Other details

We defined floating potential boundary conditions of 0 A net current through the inactive contacts, and Neumann boundary conditions of 0 A/mm^2 along the electrode shaft (except for the contacts) and head surface (except for the neck region).

We created a cube centered around the electrode contacts with a side length of 30 mm that was meshed at a higher resolution. We aligned the electrode, encapsulation layer, and cube to the contact coordinates using Rodrigues' rotation formula. The entire mesh contained 1,429,416 tetrahedral elements (head outside brain – 293,054; brain outside 30 mm cube – 834,778; brain inside 30 mm cube – 229,580; encapsulation layer – 72,004). We generated a second mesh with increased resolution to ensure solution convergence (total – 2,347,048; head

outside brain – 293,863; brain outside 30 mm cube – 1,177,452; brain inside 30 mm cube – 440,303; encapsulation layer – 435,430).

H. Nuclei segmentation

We performed manual segmentation of subcortical structures (i.e. putamen, globus pallidus externus, globus pallidus internus, subthalamic nucleus, substantia nigra, and red nucleus) using Seg3D, on the image that provided the best contrast for the nuclei of interest in the common coordinate system (Fig 4A and S3 Table). Because Seg3D doesn't permit exporting the files in the NIfTI file format, we exported the files in the nearly raw raster data (NRRD) file format, and converted to NIfTI in 3DSlicer. The resulting NIfTI file had an incorrect orientation, so we implemented the `fslorient -forceradiological` and `fslreorient2std` commands to correct this error. Tools are currently under development to automate these subcortical segmentations (Kim et al., 2014).

Due to a lack of contrast in the 1.5T and 7T T1W images, thalamic segmentation was difficult. Therefore, to define the thalamus, we used the Harvard-Oxford subcortical structural atlas distributed within FSL. Specifically, we used Cicerone to fit the thalamus to the 1.5T T1W image with 9 degrees of freedom (Miocinovic et al., 2007).

In the axial view, we used Seg3D to segment the seed and target masks used in the tractography algorithm (S3 Fig). We defined the seed mask as the white matter between the thalamus and lenticular nucleus, 1.2 mm superior to the STN. We defined two target masks, one superior to the seed mask and one inferior to the seed mask. We defined the superior target mask as the white matter between the thalamus and lenticular nucleus, 10.8 mm superior to the seed mask. We defined the inferior target mask as the cerebral peduncle of the midbrain, 17.2 mm inferior to the seed mask.

For tractography, we used an exclusion mask that included the ipsilateral thalamus, globus pallidus, putamen, CSF, and contralateral hemisphere. We used Freesurfer's `recon-all` tool

to segment the ipsilateral CSF and contralateral hemisphere masks from the 1.5T T1W image. First, we converted the output file from `recon-all` (aparc+aseg.mgz) to a NIfTI file format with Freesurfer's `mri_convert` tool. Next, we used FSL's `fslmaths` tool to extract the regions of interest from the output file with the threshold flags (`-thr`, `-uthr`) and subsequently binarized with the `-bin` flag. We then used `mri_convert -rl` to reslice this image to the original T1W image dimensions.

I. Probabilistic tractography

After we segmented the patient-specific subcortical masks, the next step was to reconstruct streamlines that would be used to define the axon trajectories. We reconstructed two sets of streamlines that represented corticofugal axons of the hyperdirect pathway and internal capsule fibers of passage. We used FSL's `bedpostx` tool to estimate the parameters for a diffusion model in each voxel (Behrens et al., 2007). Next, we used FSL's `probtrackx` tool to perform probabilistic tractography from the seed mask, with 100 streamlines generated from each seed voxel. We saved the coordinates of each streamline by passing the `-V 2` option when running `probtrackx`. One constraint was that the streamline files were generically named (i.e. particle0, particle1, etc.) for each seed voxel. Therefore, when more than one seed voxel was run with the same `probtrackx` instance, the files were overwritten with the results for each subsequent seed voxel. To save the streamlines for all voxels within the seed mask, for each voxel we ran a separate instance of `probtrackx` and concatenated the streamline files into a single file. We ran each instance of `probtrackx`, followed by the streamline processing described below, on a computational cluster to decrease the computation time.

We processed the streamlines exported from `probtrackx` to identify the streamlines that originated from the seed mask, intersected both target masks, and avoided exclusion masks and the electrode (Fig 4B). First, the streamline coordinates exported from `probtrackx` were in DW

space, so we used FSL's `img2imgcoord` tool and the DW-to-T1W transformation matrix to calculate the streamline coordinates in T1W space. We manipulated the streamline so that it formed a continuous path from the superior end of the streamline, through the seed voxel, and to the inferior end. As the streamlines were now in T1W space, we scaled the streamlines by the voxel dimensions to convert the streamlines from voxel space to millimeter space (Section D in S1 Text). Furthermore, we obtained the x-axis orientation from the sign of the determinant of the T1W image qform matrix. In our example, we multiplied the x values by -1.

Next, we checked to determine if a streamline intersected with both target masks. If so, we cropped the streamline between the target masks and if not, we excluded it from further analysis. If the cropped streamline took a trajectory above or below the superior or inferior target masks, respectively, we excluded it from further analysis. Additionally, if the cropped streamline intersected the electrode or the exclusion mask, we also excluded it from further analysis. Subsequently, we concatenated the processed streamline files for all seed voxels into the same file. In our example, 13,219 streamlines originated from the seed mask, terminated in the target masks, and avoided the electrode and exclusion mask.

J. References

- Basser PJ, Mattiello J, LeBihan D. MR diffusion tensor spectroscopy and imaging. Biophys J. 1994 Jan;66(1):259-67. PMID: 8130344.
- Fischl B. FreeSurfer. Neuroimage. 2012 Aug 15;62(2):774-81. PMID: 22248573.
- Iacono MI, Neufeld E, Akinnagbe E, Bower K, Wolf J, Vogiatzis Oikonomidis I, et al. MIDA: A Multimodal Imaging-Based Detailed Anatomical Model of the Human Head and Neck. PLoS One. 2015 Apr 22;10(4):e0124126. PMID: 25901747.
- Jenkinson M, Beckmann CF, Behrens TE, Woolrich MW, Smith SM. FSL. Neuroimage. 2012 Aug 15;62(2):782-90. PMID: 21979382.
- Kim J, Lenglet C, Duchin Y, Sapiro G, Harel N. Semiautomatic segmentation of brain subcortical structures from high-field MRI. IEEE J Biomed Health Inform. 2014 Sep;18(5):1678-95. PMID: 25192576.

- Miocinovic S, Noecker AM, Maks CB, Butson CR, McIntyre CC. Cicerone: stereotactic neurophysiological recording and deep brain stimulation electrode placement software system. Acta Neurochir Suppl. 2007;97(Pt 2):561-7. PMID: 17691348.
